# Classical Paal-Knorr Cyclization for Synthesis of Pyrrole-Based Aryl Hydrazones and In Vitro/In Vivo Evaluation on Pharmacological Models of Parkinson’s Disease

**DOI:** 10.3390/molecules30153154

**Published:** 2025-07-28

**Authors:** Maya Georgieva, Martin Sharkov, Emilio Mateev, Diana Tzankova, Georgi Popov, Vasil Manov, Alexander Zlatkov, Rumyana Simeonova, Magdalena Kondeva-Burdina

**Affiliations:** 1 Department of Pharmaceutical Chemistry, Faculty of Pharmacy, Medical University Sofia, 1000 Sofia, Bulgaria; martin.sharkov1@abv.bg (M.S.); e.mateev@pharmfac.mu-sofia.bg (E.M.); d.tsankova@pharmfac.mu-sofia.bg (D.T.); azlatkov@pharmfac.mu-sofia.bg (A.Z.); 2 Department of Internal Non-Communicable Diseases, Pathology and Pharmacology, Faculty of Veterinary Medicine, University of Forestry, 10 St. Kliment Ohridski Blvd., 1797 Sofia, Bulgaria; gpopov@ltu.bg (G.P.); vmanov@ltu.bg (V.M.); 3 Department of Pharmacology, Pharmacotherapy and Toxicology, Faculty of Pharmacy, Medical University Sofia, 1000 Sofia, Bulgaria; mkondeva@pharmfac.mu-sofia.bg

**Keywords:** synthesis, pyrrole, hydrazone, in vivo, Parkinson’s

## Abstract

Some studies performed in our laboratory on pyrrole and its derivatives pointed towards the enrichment of the evaluations of these promising chemical structures for the potential treatment of neurodegenerative conditions in general and Parkinson’s disease in particular. A classical Paal-Knorr cyclization approach is applied to synthesize the basic hydrazine used for the formation of the designed series of hydrazones (**15a**–**15g**). The potential neurotoxic and neuroprotective effects of the newly synthesized derivatives were investigated in vitro using different models of induced oxidative stress at three subcellular levels (rat brain synaptosomes, mitochondria, and microsomes). The results identified as the least neurotoxic molecules, **15a**, **15d**, and **15f** applied at a concentration of 100 µM to the isolated fractions. In addition, the highest statistically significant neuroprotection was observed for **15a** and **15d** at a concentration of 100 µM using three different injury models on subcellular fractions, including 6-hydroxydopamine in rat brain synaptosomes, tert-butyl hydroperoxide in brain mitochondria, and non-enzyme-induced lipid peroxidation in brain microsomes. The *h*MAOA/MAOB inhibitory activity of the new compounds was studied at a concentration of 1 µM. The lack of a statistically significant *h*MAOA inhibitory effect was observed for all tested compounds, except for **15f**, which showed 40% inhibitory activity. The most prominent statistically significant *h*MAOB inhibitory effect was determined for **15a**, **15d**, and **15f**, comparable to that of selegiline. The corresponding selectivity index defined **15f** as a non-selective MAO inhibitor and all other new hydrazones as selective *h*MAOB inhibitors, with **15d** indicating the highest selectivity index of >471. The most active and least toxic representative (**15d**) was evaluated in vivo on Rotenone based model of Parkinson’s disease. The results revealed no microscopically visible alterations in the ganglion and glial cells in the animals treated with rotenone in combination with **15d**.

## 1. Introduction

The appearance of generalized slowing of movements (bradykinesia) and at least one other symptom of resting tremor or rigidity in the later years of life is mostly related to the neurodegenerative disorder called Parkinson’s disease (PD). This condition is also often associated with features like loss of smell, REM behavior disorder (excessive periodic limb movements while sleeping), mood disorders, and constipation [1].

According to recent estimates, PD affects at least 1% of the population over the age of 60. It is closely related to the appearance of Lewy bodies and a decrease in dopaminergic neurons in a specific part of the brain, called the substantia nigra [1].

Parkinson’s disease may also be considered a part of the so-called synucleinopathies, along with dementia with Lewy bodies (DLB), multiple system atrophy (MSA), and pure autonomic failure (PAF), since they are related to the accumulation of an abnormal version of the protein alpha-synuclein in the nerve cells, which is also referred to as phosphorylated alpha-synuclein (P-SYN) [2].

Increasing evidence suggests that Parkinson’s disease consists of heterogeneous subtypes. Subtypes have implications for diagnosis, prognosis, and expected treatment responses. Initial subtyping focused on motor features [3,4], but recent categorizations have used data-driven clustering approaches [3]. These approaches suggest that subtypes are defined by motor and nonmotor features [3,5,6,7].

For individuals diagnosed with Parkinson’s disease, treatment focuses on symptom management, targeting both motor and nonmotor manifestations. Currently, no therapies have been proven to alter the course of the disease. Initial motor symptoms often respond to dopamine-related medications, while nonmotor symptoms typically require other types of treatments, such as antidepressants or cognitive enhancers. In cases where symptoms worsen or medications lose effectiveness, manifesting as reduced mobility between doses, persistent tremors, or involuntary movements, advanced interventions like continuous drug delivery systems or surgical options may be considered. Palliative care also plays a role in comprehensive disease management. There are many therapeutic options for treating Parkinson’s disease, including therapy with carbidopa-levodopa, monoamine oxidase-B inhibitors, and dopamine agonists [3].

Parkinson’s disease is a heterogeneous condition with both rapidly and slowly progressive forms. Its management includes pharmacologic approaches, primarily involving levodopa preparations that may be prescribed alone or in combination with other medications. Non-pharmacologic strategies are also important and encompass exercise, physical, occupational, and speech therapies. Additionally, interventions such as deep brain stimulation and levodopa-carbidopa enteral suspension can be beneficial for individuals experiencing medication-resistant tremors, symptom worsening as medication effects diminish, and dyskinesia [8]. However, currently, no pharmacologic therapies prevent or delay Parkinson’s disease progression [9].

Previous studies in our laboratory on pyrrole derivatives [10,11,12,13], along with the diverse pharmacological actions (including neurological) and low toxicity of this class of compounds, have defined them as interesting candidates for novel therapeutic agents.

The experiments indicated that at a concentration of 1 μmol, pyrrole hydrazides **11** and **12** inhibited MAOB by 26% and 50%, respectively [12]. These results suggest that the prolongation of the methylene bridge between the central pyrrole ring and the R(Ar)(C=O)-NH-NH_2_ group may be favorable for low neurotoxicity, promising neuroprotection, and relatively good MAOB inhibitory effects. A suitable way to introduce this prolongation is by using *γ*-aminobutyric acid as a condensation partner, considering the well-known CNS effects of GABA.

Our experience from the tests conducted thus far on pyrrole-based azomethine derivatives regarding their MAO inhibitory properties, along with recent data suggesting the potential effects of GABA on both motor and non-motor manifestations of Parkinson’s disease, has guided current research toward combining these two principles. This approach aims to optimize the therapeutic potential of this group of compounds.

## 2. Results

### 2.1. Synthesis of the Target Molecules

#### 2.1.1. Paal-Knorr Synthesis of the Initial Hydrazide

**Scheme 1:**
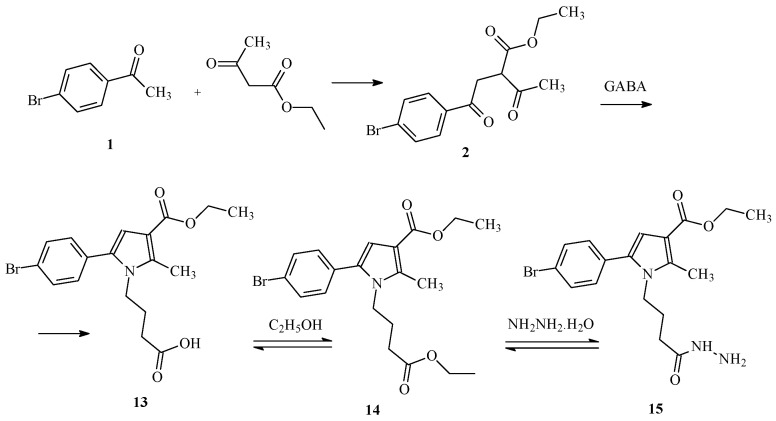
Paal-Knorr synthesis of the initial hydrazide **15**.

The Paal-Knorr pyrrole synthesis is one of the earliest and most well-known synthetic processes in organic chemistry [14]. It produces pyrroles through the condensation of a 1,4-dicarbonyl compound with an excess of primary amine, ammonia, or amino acid. The reaction usually requires acidic conditions; however, some alternative methods based on solid acid catalysts have recently been developed, which offer major advantages [15]. The application of mineral acids as acidifying components has some advantages in the laboratory, like profitability, low price, ease of use, and accessibility, which define our methodology (Scheme 1).

#### 2.1.2. General Synthesis of the Target Hydrazones, Containing Phenyl/Substituted Phenyl Carbonyl Fragment

**Scheme 2:**
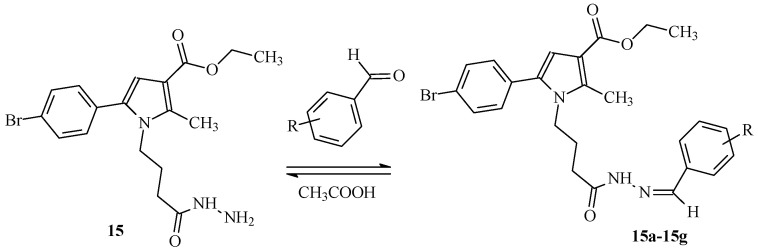
General synthesis of the target hydrazones **15a**–**15g**.

**Figure 1:**
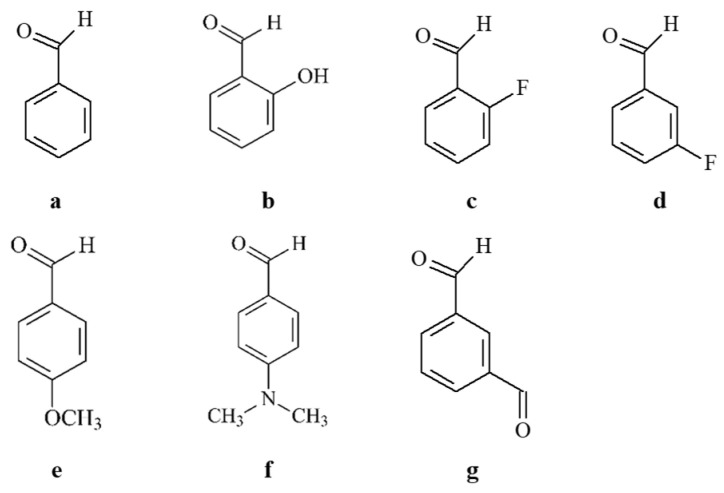
Applied phenyl/substituted phenyl aldehydes **a**–**g**.

The target Schiff bases were prepared by reacting various carbonyls (Figure 1) with GABA-based hydrazide (**15**), where an excess amount of the carbonyl partner was used (1.2 eq.) in a media of glacial acetic acid at 100 °C to complete the reaction with constant stirring (Scheme 2).

The synthesized pyrrole hydrazones were defined as stable under normal conditions. The corresponding structures of the synthesized compounds were elucidated using IR, ^1^H-NMR, and ^13^C-NMR spectral data, followed by MS data. The purity of the molecules was confirmed through the corresponding melting points, TLC characteristics, and elemental analyses. The results of the analysis confirmed the consistency of the new compounds with the expected structures.

The IR spectral results identified the presence of new signals for the valence asymmetric (*ν_as_*) vibrations of the amide NH group in the molecule of the new hydrazones at about 3200 cm^−1^ and two bands at 1688 cm^−1^ ascribable to the carbonyl C=O group (Amide I) and at 1520 cm^−1^ assigned to the deformational (*δ*) vibrations of the amide NH group (Amide II). In addition, the appearance of a band at around 813 cm^−1^ indicates the presence of a *p*-substituted phenyl residue, while the band at around 827 cm^−1^ indicates the presence of an *m*-substituted phenyl residue. The band at approximately 1660–1690 cm^−1^ is ascribed to the third position ester group (COOC_2_H_5_) in the pyrrole ring. The structural elucidation was confirmed by the relevant ^1^H- and ^13^C-NMR spectra, where the presence of the specific CH=N and CONH groups is defined by the peaks available in the range of 9.7 to 11.2 ppm and 7.9 to 11.4 ppm for ^1^H-NMR analysis and at 140.0 ppm and 168.4 ppm for the ^13^C-NMR analysis, respectively.

The corresponding IDs, melting points (m.p.), TLC characteristics, MS data, and yields are listed in Table 1. The respective IR characteristics, ^1^H-NMR, and ^13^C-NMR spectral data are presented in Section 4.

#### 2.1.3. Molecular Docking Studies Results

Additionally, a preliminary molecular docking-based in silico assessment of the influence of the methylene linker length on the binding abilities of pyrrole-containing hydrazides designed as MAOB inhibitors was conducted.

Molecular docking simulations of the binding affinity of the newly synthesized hydrazide **15** in the active site of MAOB (PDB: 2V5Z) were carried out using the docking module of Maestro–Glide. A comparison between the evaluated affinities for **15** and two representatives of this group with shorter linkers, including the glycine-based hydrazide (**11**) [16] and *β*-alanine-based hydrazide (**12**) [17], was performed. Molecular docking simulations demonstrated that the glycine-based hydrazide (**11**) obtained the best score (−9.52 kcal/mol), while the XP scores of the *β*-alanine (**12**) and *γ*-aminobutyric acid (**15**)-based hydrazides were −8.36 and −7.92, respectively. The docking values of compounds **12** and **15** as MAOB inhibitors were identical, which led to the hypothesis that pyrroles condensed with GABA could be applied as novel inhibitors against the former enzyme.

**Figure 2:**
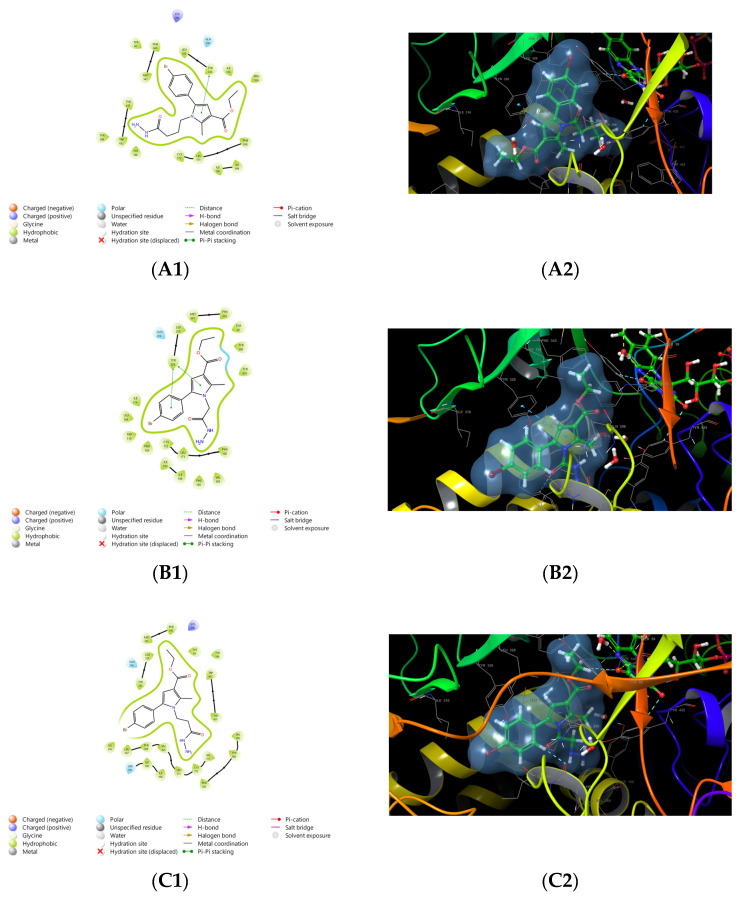
Visualizations of the intermolecular interactions of **15** within the active site of MAOB (PDB: 2V5Z): (**A1**) 2D panel; (**A2**) 3D panel, **11** with the active site of MAOB (PDB: 2V5Z); (**B1**) 2D panel; (**B2**) 3D panel and **12** with the active site of MAOB (PDB: 2V5Z); (**C1**) 2D panel; (**C2**) 3D panel.

Visualizations of the intermolecular interactions between **15**, other pyrrole-based hydrazides synthesized previously in our laboratory with shorter bridges, including one methylene group (**11**) [16] and two methylene groups (**12**) [17], and the active site MAOB are displayed in Figure 2.

Visualizations of the major intermolecular interactions showed that the glycine- and *β*-alanine-condensed pyrroles obtained identical active conformations in the active site of MAOB. Interestingly, glycine-based hydrazide **11** was stabilized by two p-p stacking interactions with the active amino acid Tyr326. Both the pyrrole moiety (4.91 Å) and the 4-bromobenze (4.59 Å) fragment participated in the interactions. The compound with two methylene linkers (**12**) was mainly stabilized by hydrophobic interactions with amino acids constructing the substrate cavity and the aromatic cage of the enzyme–Tyr60, Phe168, Val169, Leu171, Tyr398, Trp432, and Tyr435. The GABA-based pyrrole structure (**15**) formed one p-p stacking interaction with Tyr326 (4.70 Å). In addition, the hydrazide moiety was placed in the aromatic cage of MAOB (Tyr398 and Tyr435). Hydrophobic interactions with Tyr60, Pro104, Leu171, Cys172, Tyr188, Ile316, and Phe343 were also observed. Importantly, none of the examined compounds formed stable hydrogen bonds with the active pocket of MAOB.

### 2.2. Pharmacological Evaluations Results

#### 2.2.1. Neurotoxicity Assessment Results

##### Effect of Substances Administered Alone on Biomarkers Characterizing the Functional-Metabolic Profile of Brain Synaptosomes (Measured as Synaptosomal Vitality and GSH Level), Mitochondria (Measured as GSH Level and MDA Production), and Microsomes (Measured as MDA Production)

In all pharmacological evaluations, the activity of the new derivatives was compared with that of the initial *N*-pyrrolyl carboxylic acid **13**.

##### Effect of Substances Administered Alone on Biomarkers Characterizing the Functional-Metabolic Profile of Brain Synaptosomes

**Figure 3:**
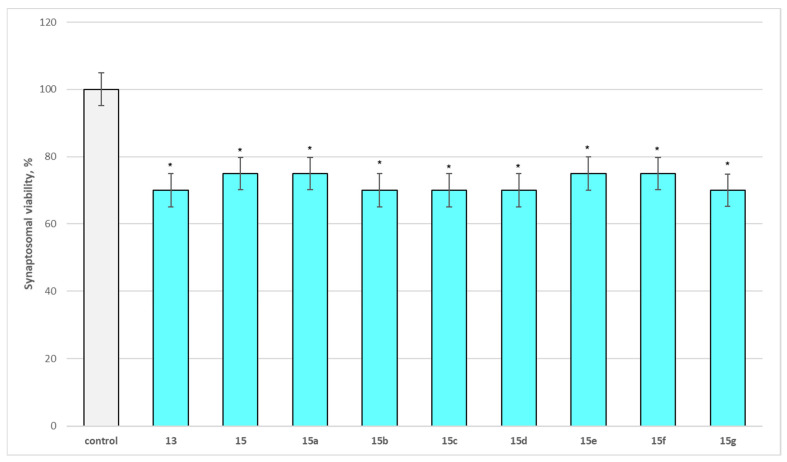
Effect of the test substances applied alone at a concentration of 100 µM on synaptosomal viability. * *p* < 0.05 vs. control (non-treated synaptosomes).

**Figure 4:**
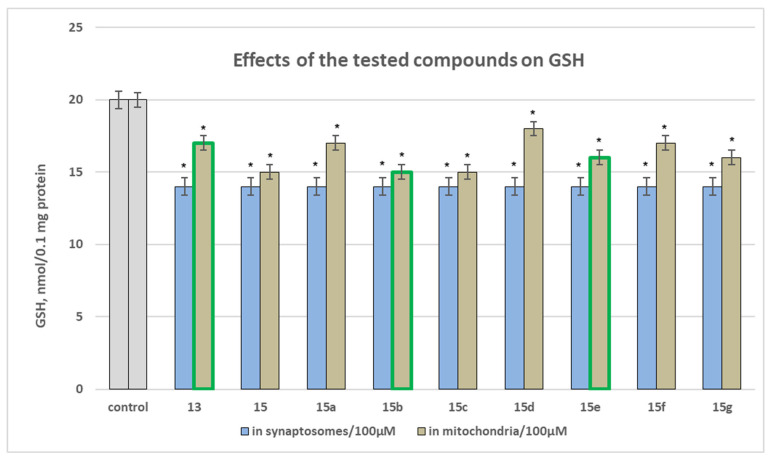
Effect of the test substances applied alone at a concentration of 100 µM on the level of GSH in isolated rat brain synaptosomes (blue color) and mitochondria (brownish color). * *p* < 0.05 vs. control (non-treated synaptosomes); * *p* < 0.05 vs. control (non-treated mitochondria).

When administered alone at a concentration of 100 µM to brain synaptosomes, the test substances (**13**, **15**, and **15a**–**15g**) exhibited a statistically significant weak neurotoxic effect compared to the control (untreated synaptosomes). They slightly decreased synaptosomal viability and reduced glutathione (GSH) levels (Figure 3 and Figure 4, respectively), and all the tested compounds revealed comparable effects.

The results showed that all evaluated substances **13**, **15**, and **15a**–**15g**, decreased synaptosomal viability statistically significant in the range of 25 to 30% and decreased the GSH level by 14% compared to the control.

##### Effect of Newly Synthesized Hydrazones, Administered Alone, on Biomarkers Characterizing the Functional-Metabolic Profile of Brain Mitochondria

When applied alone at a concentration of 100 µM to brain mitochondria, the test substances (**13**, **15**, **15a**–**15g**) exhibited a statistically significant weak neurotoxic effect relative to the control (untreated mitochondria). They slightly decreased (GSH) levels (Figure 4), where **13**, **15a**, and **15f** decreased the reduced glutathione (GSH) levels statistically significant by 15%; **15**, **15b**, and **15c**–by 25%; **15e** and **15g**–by 20% and **15d**–by 10%, compared to the control.

The data showed that **13** increased MDA production statistically significantly by 79%; **15**–by 73%; **15a**–increased by 59%; **15b**–increased by 99%; **15c**–by 110%; **15d**–by 57%; **15e**–by 119%; **15f**–by 69%, and **15g**–by 143%, compared to the control.

The obtained results identified the substances with the weakest neurotoxic effect, derivatives **15a**, **15d**, and **15f**. The effects of these substances were compared with those of *N*-pyrrolyl carboxylic acid (**13**).

##### Effect of Newly Synthesized Hydrazones, Administered Alone, on Biomarkers Characterizing the Functional Metabolic Profile of Brain Microsomes

**Figure 5:**
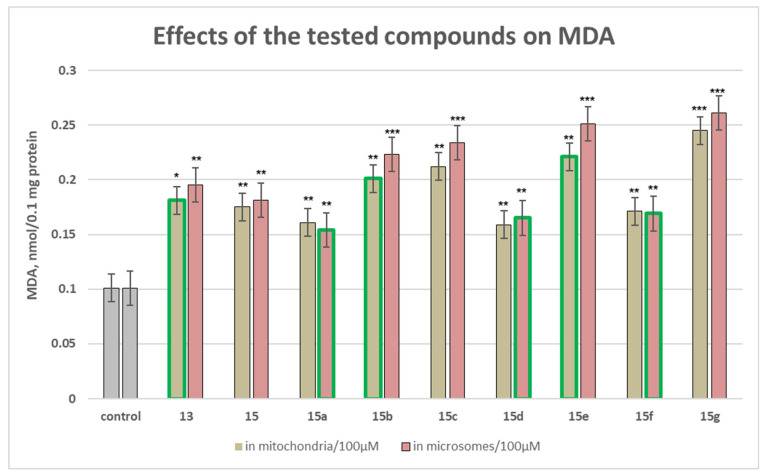
Effect of test substances applied alone at a concentration of 100 µM on MDA production in isolated rat brain mitochondria (brownish color) and rat brain microsomes (rufescent color). * *p* < 0.05; ** *p* < 0.01; *** *p* < 0.001 vs. control (non-treated mitochondria). ** *p* < 0.01; *** *p* < 0.001 vs. control (non-treated microsomes).

When self-administered at a concentration of 100 µM to brain microsomes, the test substances (**15**, **15a**–**15g**) showed statistically significant weak neurotoxic effects relative to the control (untreated microsomes). They slightly increased the marker of lipid peroxidation, indicated as production of malondialdehyde (MDA) (Figure 5), with **13** increasing MDA production statistically significantly by 93%; **15a**–by 52%; **15b**–by 121%; **15c**–by 132%; **15d**–by 63%; **15e**–by 149%; **15f**–by 67% and **15g**–by 158%, compared to the control.

The data indicated that the weakest neurotoxic effects were manifested by **15a**, **15d**, and **15f**. The effects of the target compounds were compared to those of the initial *N*-pyrrolyl carboxylic acid (**13**).

##### Effects of the Target New Compounds **13**, **15**, **15a**–**15g** on Human Recombinant MAOA/MAOB Enzyme Results

**Figure 6:**
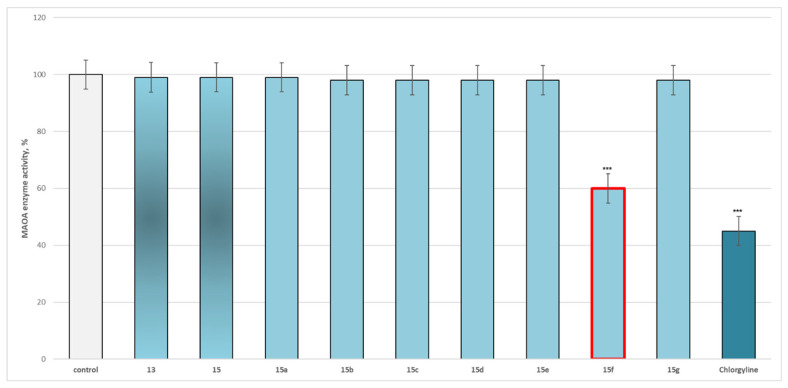
Effect of newly synthesized hydrazones (at a concentration of 1 µM) on the activity of human recombinant MAOА enzyme (*h*MAOA). *** *p* < 0.001 vs. control (pure *h*MAOA).

**Figure 7:**
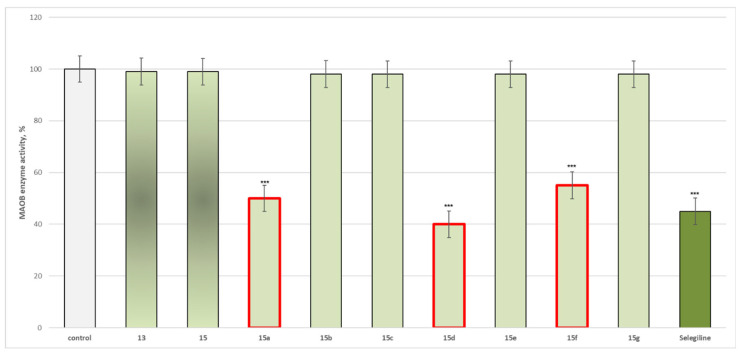
Effect of newly synthesized hydrazones (at a concentration of 1 µM), on the activity of human recombinant MAOB enzyme (*h*MAOB). *** *p* < 0.001 vs. control (pure *h*MAOB).

All newly synthesized compounds were screened for potential MAOA and/or MAOB inhibitory effects. The corresponding results are presented in Figure 6 and Figure 7, respectively.

The results from the assessment showed that from all tested compounds, only **15f** exhibited a statistically significant MAOA inhibitory effect by decreasing the enzyme activity by 40% compared to the control (pure *h*MAOA). This value was similar to that of chlorgyline, which showed 55% inhibitory activity (Figure 6).

The evaluation of MAOB activity indicated that three representatives: **15a**, **15d**, and **15f**, exhibited statistically significant MAOB inhibitory effects, decreasing the enzyme activity by 50%, 60%, and 45%, respectively, compared to the control (pure *h*MAOB). The effects were comparable to those of Selegiline, which inhibited the enzyme by 55% (Figure 7), with **15d** showing even better MAOB-inhibiting activity than Selegiline.

The corresponding selectivity index IC_50_ (EC_50_) of the representatives showing MAOA and/or MAOB inhibitory effect was found to identify the best *h*MAOB selective molecule (Table 2).

SI: *h*MAOB selectivity index = IC_50_(*h*MAOA)/IC_50_(*h*MAOB) (1)

The results of the conducted studies indicate that the best *h*MAOB selectivity index is relative to compounds **15a** (>289) and **15d** (>471). The data determined compound **15f** to be a non-selective MAO inhibitor (1.16). Compound **15d** exhibited the highest *h*MAOB selectivity index and was tested in an in vivo Rotenone-induced model of Parkinsonism in mice for possible neuroprotection.

#### 2.2.2. Neuroprotection Effects Results

The substances characterized by the upfront data as least neurotoxic and exhibiting the highest MAOB inhibitory effect were assessed for their possible neuroprotective properties in three models of induced neurotoxicity: a model of 6-OHDA-induced neurotoxicity in isolated rat brain synaptosomes, a model of *t*-BuOOH-induced oxidative stress in isolated rat brain mitochondria, and a model of non-enzyme-induced lipid peroxidation (Fe^2+^/AA) in isolated rat brain microsomes.

##### Effect of Most Active and Least Toxic Substances in a Model of 6-OHDA-Induced Neurotoxicity on Parameters Characterizing the Functional-Metabolic Profile of Brain Synaptosomes

**Figure 8:**
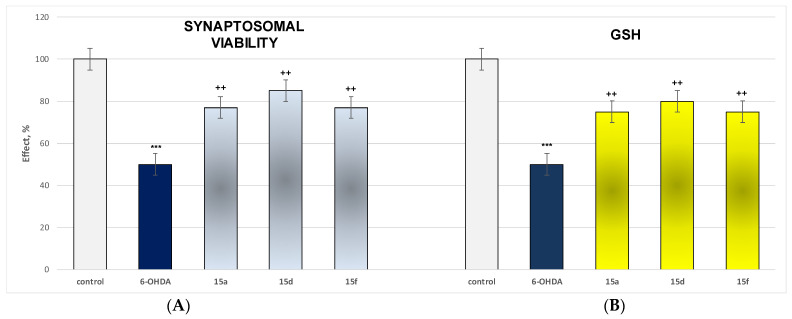
Effect of the most active and least toxic substances, in combination with 6-OHDA, on synaptosomal viability (**A**) and level of reduced glutathione (GSH) (**B**). *** *p* < 0.001 vs. control (non-treated synaptosomes); ++ *p* < 0.01 vs. 6-OHDA.

The toxic agent (6-OHDA), applied alone at a concentration of 150 µM to isolated synaptosomes, resulted in a statistically significant 50% decrease in synaptosomal viability and GSH levels compared to the control (untreated synaptosomes) (Figure 8).

When combined with 6-OHDA, all evaluated substances (at a concentration of 100 µM) exhibited a statistically significant neuroprotective effect against the toxic agent (Figure 8) for both evaluated parameters.

The best neuroprotective effect was expressed by compound **15d**, preserving synaptosomal viability statistically significant by 15% and GSH level statistically significant by 20% compared to the toxic agent (6-OHDA).

##### Effect of the Most Active and Least Toxic Substances in a Model of *t*-BuOOH-Induced Oxidative Stress on Parameters Characterizing the Functional-Metabolic Profile of Brain Mitochondria

**Figure 9:**
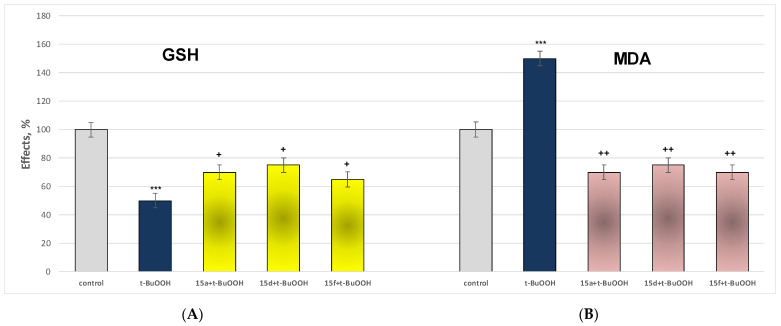
Effect of the most active and least toxic substances, in combination with *t*-BuOOH, on GSH level (**A**) and on MDA production (**B**). *** *p* < 0.001 vs. control (non-treated mitochondria); + *p* < 0.01 vs. *t*-BuOOH; ++ *p* < 0.01 vs. *t*-BuOOH.

In isolated brain mitochondria, *t*-BuOOH applied alone statistically significantly decreased GSH levels by 50% and increased MDA production by 150% compared to the control (untreated mitochondria) (Figure 9).

When combined with *t*-BuOOH, the evaluated substances (at a concentration of 100 µM) exhibited a statistically significant neuroprotective effect compared to the toxic agent, with **15d** showing the most pronounced neuroprotection among the parameters studied (Figure 9).

##### Effect of Most Active and Least Toxic Substances in a Model of Non-Enzyme-Induced Lipid Peroxidation (Fe^2+^/AA) on Parameters Characterizing the Functional Metabolic Profile of Brain Microsomes

**Figure 10:**
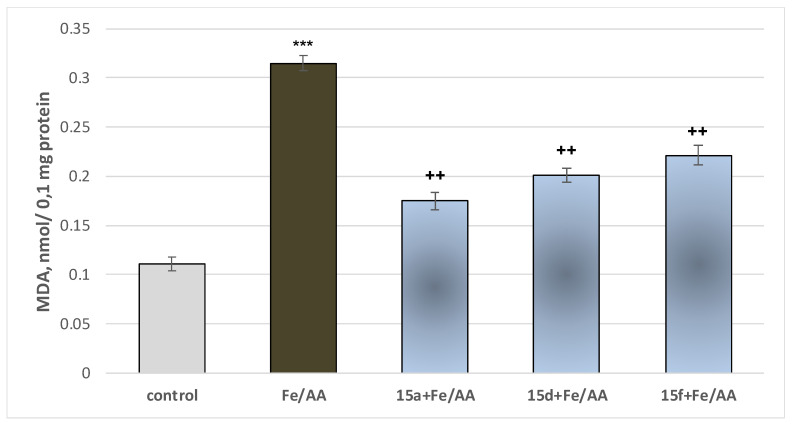
Effect of the most active and least toxic substances under conditions of non-enzyme-induced lipid peroxidation (Fe^2+^/AA). *** *p* < 0.001 vs. control (non-treated microsomes); ++ *p* < 0.05 vs. Fe^2+^/AA.

Incubation of brain microsomes with Fe^2+^/AA resulted in a statistically significant 284% increase in MDA production relative to that in the control (untreated microsomes) (Figure 10).

All tested substances (at a concentration of 100 µM) in combination with Fe^2+^/AA showed a statistically significant antioxidant effect against the toxic agent, with **15a** exhibiting the most prominent effect on MDA production (Figure 10).

### 2.3. Histopathological Analysis Results

The histological morphology of the mouse brains, mainly the areas of the cerebellar cortex and cerebellum, was examined microscopically.

**Figure 11:**
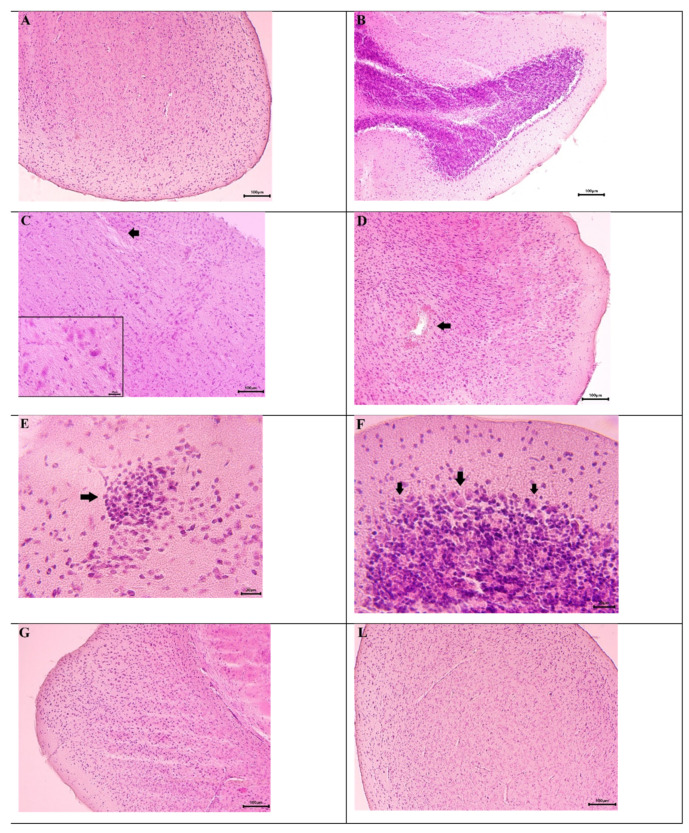
Histological examination of mouse brains (H&E). (**A**) Control group of animals: normal histoarchitectonics of the cerebral cortex; (**B**) Control group of animals: normal histological structure of the cortex of cerebellum; (**C**) Rotenone-treated animal: loss of intercellular space integrity (arrow) and pyknotic changes in ganglion cells (high magnification); (**D**) Rotenone-treated animal: degenerative-necrotic area with hemorrhage in brain tissue (arrow); (**E**) Rotenone-treated animal: glial nodule in brain parenchyma (arrow); (**F**) Cerebellum of rotenone-treated animal: pyknotic changes in Purkinje cells (arrows); (**G**) Animal treated with **15d** alone: normal brain structure; (**L**) Animal treated with combination of **15d** with rotenone: unaltered brain structure.

Light microscopy revealed no alterations in the ganglion and glial cells of the brain structures in the control group. The cerebral cortex had a normal layered structure without a sharp boundary between the individual layers (Figure 11A). The cortical neurons had a normal histological appearance, possessing oval nuclei and pale cytoplasm. Astrocytes were found in the neuropil without evidence of pathological changes, and the blood vessels had an intact perivascular space. The histological structure of the cerebellum showed no visible alterations. The cortical cerebellar zone had a well-defined layered guard with clearly defined outer molecular, middle Purkinje, and inner granule layers (Figure 11B).

In the cerebral cortex of animals treated with rotenone, distinct degenerative-necrotic changes were observed, expressed as shrunken neurons with intensely eosinophilic dark-stained cytoplasm and pyknotic nuclei. Foci with loss of normal integrity and vacuolization of the neuropil were also observed (Figure 11C). Hemorrhages were observed in separate areas of the brain parenchyma (Figure 11D). Glial nodules were found in the cerebellar cortex, mostly in the peripheral zones (Figure 11E). In the cerebellum, the main changes were detected in the layer of Purkinje cells. The observed alterations were mainly of a degenerative nature. The Purkinje cells were deformed, with loss of their characteristic pyriform shape, appeared shrunken with an irregular appearance, and contained dark-stained cytoplasm and barely discernible nucleoli (Figure 11F). The linear arrangement was disturbed, with marked disposition in individual areas.

In the brains of mice treated alone with **15d**, no microscopically visible changes were found, and the histological architecture was similar to that of the control group (Figure 11G).

Light microscopy of the brains from mice treated with rotenone in combination with **15d** revealed preserved histoarchitectonics (Figure 11L). No microscopic alterations were found in the ganglion and glial cells. Single glial nodules were observed in separate areas of the brain.

## 3. Discussion

The well-known intermolecular cyclocondensation reaction of Paal-Knorr, based on the interaction of primary amino acids and 1,4-dicarbonyl compounds, is still the most commonly applied approach for de novo pyrrole synthesis. A number of mono- and poly-substituted pyrroles and pyrrole-based acids, including *β*-trifluoromethylpyrroles, tetraarylpyrrole fluorophores, atorvastatin lactone, 1,3′-bipyrroles, 1-acylaminopyrroles, pyrrole-fused azepines etc. [18] are obtained using this approach. In addition, a variety of different acid resin catalysts have been investigated in an attempt to optimize the approach [18].

An interesting pyrrole cyclocondensation for the preparation of fused pyrroles was reported by Yasmin and Ray, based on the preparation of a doubly activated diene via a Heck reaction with methyl acrylate, followed by treatment with benzylamine to give a fused pyrrole in 93% yield [19]. In another study, a number of metal-mediated cyclization approaches for the synthesis of 2,5-disubstituted pyrroles were mentioned, based on the interaction of primary amines and unsaturated 4-carbon substrates [20]. Skrydstrup and coworkers performed similar double hydroamination reactions using an Au(I) catalyst [21]. The Demir group used an Au(I)/Zn(II) catalyst system to prepare 2-aminopyrroles via the hydroamination/cyclization of 4-yne nitriles [22,23]. Xi and colleagues investigated double cross-coupling reactions between 1,4-diiodo-1,3-dienes and anilines in the synthesis of substituted pyrroles [24].

Some experiments performed in our laboratory have shown that the application of classical acidification of the reaction media with mineral or organic acid and/or MW irradiation [13,16,17,25], when obtaining structural analogs of defined *N*-pyrrolylcarboxylic acids, is advantageous as it is environmentally friendly, inexpensive, and does not require expensive catalysts and/or specific equipment and operating conditions. These specificities defined the application of classical Paal-Knorr cyclocondensation as the main approach for synthesizing the initial *N*-pyrrolyl carbohydrazide. The target molecules were obtained in good reaction times and with acceptable yields.

### 3.1. Molecular Docking Studies

The developments that advanced information technology have defined computational approaches as an important component of modern drug discovery science.

The possibility of reliably modeling the tertiary structure of proteins using amino acid sequences to help infer their molecular functions, along with the availability of confidently predicting the putative ligand-binding pockets from computer-generated protein models for further use as target site identification, has become a good practice in the identification of novel lead compounds [26].

The observed interactions and binding distances suggest that the interaction of the shortest hydrazide **11** was stabilized by two p-p stacking interactions. For the longer hydrazide **12**, hydrophobic interactions with the amino acids constructing the substrate cavity and the aromatic cage of the enzyme were the main stabilizing forces. For the longest hydrazide **15**, one π-π stacking interaction with Tyr326 was observed. In addition, it is worth mentioning that the hydrazide moiety was placed in the aromatic cage of MAOB, while none of the examined compounds formed stable hydrogen bonds with the active pocket of the enzyme.

Some previous studies indicated that at a concentration of 1 μmol, pyrrole hydrazides **11** and **12** inhibit MAOB by 26% and 50%, respectively [12]. These results suggested that the prolongation of the methylene bridge between the central pyrrole ring and the R(Ar)(C=O)-NH-NH_2_ group may be considered favorable for performance of low neurotoxicity, promising neuroprotection, and relatively good inhibitory MAOB effects. Thus, by synthesizing hydrazide **15**, our study aimed to evaluate the addition of two more methylene groups in the bridge, expecting to improve the binding capacity of the final hydrazide. The obtained results identified that the additional extension of the methylene bridge leads to a decrease and/or loss of MAOB inhibitory activity with low or no effect on neurotoxicity.

### 3.2. Pharmacological Evaluations

Parkinson’s disease is a progressive neurodegenerative disorder characterized by reduced dopamine production due to the loss of dopamine-producing neurons in the brain. While there is no cure, treatment focuses on symptom control using medications such as MAO-B inhibitors, dopamine agonists, and levodopa, alone or in combination. Levodopa is the most effective but can lead to long-term complications, so its use is often delayed, especially in younger patients [27,28,29].

Research comparing these treatments has shown varied effectiveness and side effects, with some dopamine agonists ranking higher than MAO-B inhibitors. One study found that patients using dopamine agonists had a lower risk of death than those using MAO-B inhibitors, although further research is needed due to the small sample sizes [27,28,30].

Delaying the initiation of levodopa therapy by starting with alternative therapies may reduce the risk of motor complications. As treatment options expand, both medical and surgical approaches continue to evolve, offering improved management of the disease [31].

#### 3.2.1. Neurotoxicity Assessment

A group of subcellular fractions may be used for the preliminary neurotoxicity evaluation of newly synthesized molecules. These fractions most often include synaptosomes, mitochondria, and microsomes. However, synaptosomal, microsomal, and mitochondrial dysfunction are well-established contributors to PD, with synaptic deficits frequently observed in PD and linked to the appearance of motor and cognitive symptoms. Synaptosomes, with their unique composition and role in neurotransmission, are increasingly recognized as crucial sites of pathology in Parkinson’s disease and can disrupt neurotransmitter release, synaptic plasticity, and neuronal communication [32].

Mitochondrial dysfunction plays a recurring role in neurodegenerative diseases and involves multiple interconnected defects, such as disruptions in calcium and iron balance, energy production, and oxidative stress, making it challenging to address as a single target. Some investigations have defined that exposure to specific neurotoxins may lead to acute parkinsonian syndrome that is clinically indistinguishable from PD [33]. In addition, microsomal dysfunction, particularly in the context of mitochondria, is strongly implicated in the development and progression of several neurodegenerative diseases.

Thus, identifying the possible toxicity of the new derivatives towards mitochondria and other sub-cellular fractions is important for the development of new leading central nervous system (CNS)-acting agents.

The performed experiments showed that all target molecules showed statistically significant weak neurotoxic effects, relative to the control (the corresponding untreated synaptosomes, mitochondria, and microsomes), with **15a**, **15d**, and **15f** identified as the molecules with the least in vitro neurotoxicity on the evaluated parameters characterizing the functional metabolic status of the evaluated sub-cellular fractions.

#### 3.2.2. Effects of the Target New Compounds **13**, **15**, **15a**–**15g** on Human Recombinant MAOA/MAOB Enzyme

Pharmacological treatment for Parkinson’s disease primarily targets motor symptoms using dopamine-based therapies. The initial treatment options include levodopa, dopamine agonists, and MAOB inhibitors. Levodopa provides the most effective symptom control but may lead to dyskinesias with long-term use. Dopamine agonists and MAOB inhibitors offer milder symptom relief but have different side effect profiles [9].

Dopamine agonists are associated with impulse control disorders in a significant proportion of patients, and withdrawal can be difficult due to dependence-like symptoms. Anticholinergic medications may help with tremors in younger patients but have cognitive risks [34].

Over time, many patients use a combination of medications to maximize benefits and minimize the side effects. Adjuncts like COMT inhibitors and additional doses of MAO-B inhibitors can enhance the effect of levodopa [35,36].

Advanced therapies, such as deep brain stimulation, focused ultrasound, and levodopa-carbidopa enteral suspension, are options for patients with motor complications that are not well managed by medications alone [37].

Non-motor symptoms are generally treated with non-dopaminergic drugs. MAOB inhibitors help sustain motor function by blocking dopamine breakdown and are the focus of ongoing research into more targeted, effective therapies with fewer side effects.

Current investigations on the link between MAOA and MAOB gene polymorphisms and Parkinson’s disease susceptibility have produced conflicting results. Some studies suggest a possible link between non-motor Parkinson’s symptoms and MAOA, but the results are not conclusive. In addition, some other results imply that it is not MAOB but MAOA that contributes to DA metabolism [38].

Classically, it is believed that MAOA and MAOB have differential substrate selectivity, with epinephrine, norepinephrine, melatonin, and serotonin being metabolized by MAOA, while phenylethylamine and benzylamine are degraded by MAOB [39].

In addition, recent research has presented some evidence that MAOA, not MAOB, is mainly responsible for mediating DA degradation [40].

This necessitated the investigation of the possible MAO-inhibiting effects of the new molecules and the determination of the selectivity towards the corresponding isoform. Thus, the evaluations performed on the MAO effects of the new *N*-pyrrolyl hydrazide-hydrazones **15a**–**15g** showed that **15f** exhibited a statistically significant MAOA inhibitory effect and **15a**, **15d**, and **15f** exhibited statistically significant МАОВ inhibitory effects. The data determined compound **15f** to be a non-selective MAO inhibitor, while **15d** exhibited the highest *h*MAOB selectivity index. The latter was additionally tested in an in vivo Rotenone-induced model of Parkinsonism in mice for possible neuroprotection.

#### 3.2.3. Neuroprotection Effects

Several hypotheses have been proposed to explain the complex and still not identified mechanism of the pathology of Parkinson’s disease (PD). It is believed that genetic and environmental factors affect neurodegenerative diseases, with oxidative stress being important. A postmortem analysis of brain tissues revealed the appearance of lipid peroxidation products, carbonyl modifications of soluble proteins, and oxidation products of DNA and RNA. Therefore, the application of in vitro animal models of oxidative stress using reagents like 1-methyl-4-phenyl-1,2,3,6-tetrahydropyridine (MPTP), rotenone, 1,1′-dimethyl-4,4′-bipyridinium dichloride (paraquat), and 6-hydroxydopamine (6-OHDA) mimicking this condition is a well-known procedure for evaluating the grounds of this hypothesis [41].

#### 3.2.4. Effect of Most Active and Least Toxic Substances in a Model of 6-OHDA-Induced Neurotoxicity on Parameters Characterizing the Functional-Metabolic Profile of Brain Synaptosomes

6-OHDA represents an in vitro model resembling the neurodegenerative processes observed in Parkinson’s disease. The metabolism associated with the oxidation of 6-OHDA leads to the formation of reactive oxygen species (ROS) and reactive quinones. These induce dopamine neurotoxicity and neurodegeneration [41]. In this relation, synthesizing a molecule that potentially suppresses the formation of these reactive species will prevent neuronal cells from oxidative damage. In response, we screened for the neuroprotective effects of newly synthesized azomethine derivatives. The results from our experimental assessment indicated that among all evaluated compounds, **15d** showed statistically significant neuroprotective effects on both evaluated parameters.

#### 3.2.5. Effect of the Most Active and Least Toxic Substances in a Model of *t*-BuOOH-Induced Oxidative Stress on Parameters Characterizing the Functional-Metabolic Profile of Brain Mitochondria

The changes in the physiology of the cellular mitochondria, related to the depletion of reduced glutathione and oxidation of SH groups of key enzymes in this compartment, together with alterations in the integrity of the mitochondrial membrane, are the two possible mechanisms related to *t*-BuOOH toxicity. Both reactions result in the induction of lipid peroxidation, which can lead to oxidative changes in the cell and subsequent neurodegeneration [42,43,44]. These variations are suitable for using this model for the in vitro evaluation of the probable neuroprotective effects of the molecules.

In this toxicity model, **15d** showed the most pronounced neuroprotection among the parameters studied. The reduced GSH levels and the availability of the molecule to scavenge free radicals produced by *t*-BuOOH metabolism are probably the reasons for the optimal results.

#### 3.2.6. Effect of Most Active and Least Toxic Substances in a Model of Non-Enzyme-Induced Lipid Peroxidation (Fe^2+^/AA) on Parameters Characterizing the Functional-Metabolic Profile of Brain Microsomes

The microsomal fraction, isolated through differential centrifugation, includes membrane components derived from the intracellular structures. This fraction has been used as a model system in studies investigating lipid oxidation and assessing the antioxidant potential of various bioactive compounds [45].

The most well-known ROS in PD are hydroxide ions, superoxide anions, and hydrogen peroxide, which induce neurodegeneration and lipid peroxidation processes related to high Fe and Ca content in the substantia nigra. Thus, we assessed the changes in the MDA production under non-enzyme-induced lipid peroxidation Fe^2+^/AA model and determined that the most prominent effect on MDA production was performed by **15a**.

## 4. Materials and Methods

### 4.1. Chemistry

The purity of the obtained compounds and the progress of the reactions were controlled by thin-layer chromatography (TLC) on TLC Silicagel 60 F_254_ plates, using CHCl_3_:C_2_H_5_OH = 10:1 *v*/*v* as a mobile phase (eluent). Melting points were determined in open capillary tubes using an IA 9200 ELECTROTHERMAL apparatus (Electrothermal Engineering, Southend-on-Sea, England). All chemical names are given according to IUPAC using the ChemBioDraw Ultra software, Version 11.0, CambridgeSoft. The IR spectra were obtained in the range of 400–4000 cm^−1^ on a Nicolet iS10 FT-IR spectrophotometer using the ATR technique with a Smart iTR adapter. ^1^H-NMR spectra were recorded on a Bruker-Spectrospin WM400 MHz, Faelanden, Switzerland, operating at 400 MHz, as *δ* (ppm) relative to TMS as the internal standard. Mass spectra were registered on a 6410 Agilent LCMS triple quadrupole mass spectrometer (LCMS) with an electrospray ionization (ESI) interface. Elemental analyses were performed using a Euro EA 3000-Single, EUROVECTOR Sp Aanalyser. All chemicals and reagents used as starting materials were purchased from Sigma-Aldrich and Fluka and used without purification.

#### 4.1.1. Paal-Knorr Cyclization for Synthesis of the Target N-Pyrrolyl Carboxylic Acid

The condensation of the 1,4-dicarbonyl compound ethyl 2-acetyl-4-(4-bromophenyl)-4-oxobutanoate (**2**) was obtained according to a procedure reported by Bijev et.al. [46]. Next the required 4-(5-(4-bromophenyl)-3-(ethoxycarbonyl)-2-methyl-1H-pyrrole-1-yl)-butanoic acid (*N*-pyrrolylcarboxylic acid (**13**)) was synthesized through a classical Paal-Knorr cyclization of 0.1 mol ethyl 2-acetyl-4-(4-bromophenyl)-4-oxobutanoate (**2**) and 0.14 eq. of gamma aminobutyric acid (GABA), dissolved in sufficient ethanol and acidified with 36% hydrochloric acid. The mixture was heated on a stirrer for 75 min.

4-(5-(4-Bromophenyl)-3-(ethoxycarbonyl)-2-methyl-1H-pyrrole-1-yl)butanoic acid (*N*-pyrrolyl carboxylic acid) (**13**)

Yield: 88%; m.p. 93.4–94.8; IR (cm^−1^): 3345 (OH), 1771 (C=O),1688 (COOC_2_H_5_), 1244 (C-O), 834 (p-substituted C_6_H_4_), 547 (C-Br); ^1^H-NMR (*δ*H, 400 MHz, DMSOd_6_): 1.26 [t, 3H, CH_2_CH_3_], 1.61–1.69 [m, 2H, CH_2_CH_2_CH_2_], 2.1 [t, 2H, CH_2_CH_2_CH_2_], 2.53 [s, 3H, CH_3_(2)], 4.15–4.20 [m, 2H, CH_2_CH_2_CH_2_], 3.95 [t, 2H, CH_2_CH_3_], 6.43 [s, 1H, H(4)], 7.35–7.37 [m, 2H, H(3′), H(5′)], 7.62–7.64 [m, 2H, H(2′), H(6′)], 12.4 [s, 1H, OH]; ^13^C-NMR (100 MHz, DMSOd6): δ 177.3, 164.8, 137.4, 135.9, 132.1 (2C), 131.02, 134.04, 121.2, 120.9, 110.3, 111.9, 59.3, 43.4, 30.7, 25.7, 14.1 and 11.7; LC-MS (ESI): [M+H]^+^ 395.26.; Anal. Calc. for C_18_H_20_BrNO_4_: C, 54.84; H, 5.11, Br, 20.27, N, 3.55; O, 16.23. Found: C, 53.84; H, 5.23, Br, 21.27, N, 3.42.

#### 4.1.2. Synthesis of the Intermediate N-Pyrrolyl Carboxylic Ester

The obtained *N*-pyrrolyl carboxylic acid undergoes a modified esterification carried out for obtaining the intermediate ethyl 5-(4-bromophenyl)-1-(1-ethoxy-1-oxobutal-4-yl)-2-methyl-1H-pyrrole-3-carboxilate (**14**). SOCl_2_ (0.1 mol) was added dropwise to *N*-pyrrolylcarboxylic acid (**13**) (0.05 mol) diluted in absolute ethanol at 0 °C. After 60 min, the reaction mixture was heated using a stirrer for 120 min. The solvent was removed, and the obtained oil was washed with Na_2_CO_3_. The final product was incorporated into the next synthetic phase without isolation.

#### 4.1.3. Synthesis of the Initial Hydrazide

The necessary hydrazide ethyl 5-(4-bromophenyl)-1-(4-hydrazinyl-4-oxobutyl)-2-methyl-1H-pyrrole-3-carboxylate (**15**) was synthesized through hydrazinolysis, conducted with 0.04 mol of ethyl ester (**14**) and 0.16 mol hydrazine hydrate (64%) dissolved in absolute ethanol and refluxed under constant stirring in a round-bottom flask for 6 h. The hydrazide was then filtered after cooling and washed with ethanol. Recrystallization in ethanol was performed for purification.

Ethyl 5-(4-bromophenyl)-1-(4-hydrazinyl-4-oxobutyl)-2-methyl-1H-pyrrole-3-carboxylate (**15**).

Yield: 81%; m.p. 99.8–101.5; IR (cm^−1^): 3367 (NH), 2982 (CH_3_ and CH_2_), 1684 (COOC_2_H_5_), 1666 (Amide I), 1643 (Amide II), 1252 (C-O), 1073 (C-N), 812 (*p*-substituted C_6_H_4_), 546 (C-Br); ^1^H-NMR (*δ*H, 400 MHz, DMSOd_6_): 1.27 [s, 3H, CH_2_CH_3_], 2.06–2.15 [m, 2H, NH_2_], 2.28 [s, 2H, CH_2_CH_2_CH_2_], 2.53 [s, 2H, CH_2_CH_2_CH_2_], 3.56 [s, 3H, CH_3_(2)], 3.93–3.97 [m, 2H, CH_2_CH_3_], 4.16–4.20 [m, 2H, N(CH_2_)], 6.43 [s, 1H, H(4)], 7.35–7.55 [m, 2H, H(3′), H(5′)], 7.62–7.64 [m, 2H, H(2′), H(6′)], 7.79–7.91 [m, 1H, NH-N]; ^13^C-NMR (100 MHz, DMSOd_6_): *δ* 177.3, 164.0, 137.7, 135.9, 132.1 (2C), 131.2 (2C), 131.04, 121.3, 110.7, 110.3, 59.3, 43.4, 39.4, 25.7, 14.9 and 11.7; LC-MS (ESI): [M+H]^+^ 409.30.; Anal. Calc. for C_19_H_22_BrN_3_O_3_: C, 52.95; H, 5.43, Br, 19.57, N, 10.19; O, 11.76. Found: C, 51.95; H, 5.29, Br, 20.57, N, 10.25.

#### 4.1.4. General Synthesis of the New Hydrazones **15a**–**15g**

The corresponding *N*-pyrrolyl-carbohydrazide **15** (1 mmol) and an excess amount of 1.2 equivalents of any of the carbonyl compounds **a**, **b**, **c**, **d**, **e**, **f**, or **g** (Figure 1) were incubated in a glacial acetic acid in a 25 mL round-bottomed flask and stirred at 100 °C to complete the reaction under TLC control. The obtained products were isolated, washed with diethyl ether, and re-crystallized, where necessary, using ethanol.

(E)-Ethyl 1-(4-(2-benzylidenehydrazinyl)-4-oxobutyl)-5-(4-bromophenyl)-2-methyl-1H-pyrrole-3-carboxylate (**15a**).

Yield: 83%; m.p. 110.0–110.9; IR (cm^−1^): 3191 (NH), 2976 (CH_3_ and CH_2_), 1693 (COOC_2_H_5_), 1667 (Amide I), 1598 (Amide II), 1246 (C-O), 815 (p-substituted C_6_H_4_), 544 (C-Br); ^1^H-NMR (δH, 400 MHz, DMSOd_6_): 1.26 [s, 3H, CH_2_CH_3_], 2.10 [s, 2H, CH_2_CH_2_CH_2_], 2.35 [s, 2H, CH_2_CH_2_CH_2_], 3.2 [s, 3H, CH_3_(2)], 4.17 [s, 2H, N(CH_2_)], 4.3 [s, 2H, CH_2_CH_3_], 6.4 [s, 1H, H(4)], 7.4 [s, 3H, H(3″), H(4″), H(5″)], 7.6 [s, 2H, H(3′), H(5′)], 7.72–7.8 [m, 2H, H(2′), H(6′)], 7.88–7.91 [m, 2H, H(2″), H(6″)], 9.8 [s, 1H, NH-N], 11.2 [s, 1H, CH=N]; ^13^C-NMR (100 MHz, DMSOd_6_): *δ* 173.6, 168.4, 144.1, 142.1, 133.7, 132.04 (3C), 132.0, 131.2, 129.3 (2C), 128.3 (4C), 131.04, 110.7, 59.3, 43.6, 39.7, 39.4, 14.9 and 11.7; LC-MS (ESI): [M+H]^+^ 497.40.; Anal. Calc. for C_25_H_26_BrN_3_O_3_: C, 60.49; H, 5.28, Br, 16.10, N, 8.47; O, 9.67. Found: C, 61.95; H, 5.29, Br, 15.97, N, 9.25.

(E)-Ethyl 5-(4-bromophenyl)-1-(4-(2-(2-hydroxybenzylidene)hydrazinyl)-4-oxobutyl)-2-methyl-1H-pyrrole-3-carboxylate (**15b**).

Yield: 87%; m.p. 119.5–120.3; IR (cm^−1^): 3424 (OH), 3279 (NH), 2975 (CH_3_ and CH_2_), 1693 (COOC_2_H_5_), 1620 (Amide I), 1571 (Amide II), 1249 (C-O), 832 (p-substituted C_6_H_4_), 526 (C-Br); ^1^H-NMR (*δ*H, 400 MHz, DMSOd_6_): 1.25 [s, 3H, CH_2_CH_3_], 2.02 [t, 2H, CH_2_CH_2_CH_2_], 2.44 [s, 3H, CH_3_(2)], 2.50 [s, 2H, CH_2_CH_2_CH_2_], 4.19 [s, 2H, N(CH_2_)], 4.2 [s, 2H, CH_2_CH_3_], 6.4 [s, 1H, H(4)], 7.0 [s, 2H, H(3″), H(5″)], 7.56 [s, 1H, H(4″)), 7.68 [s, 2H, H(3′), H(5′)], 7.69 [s, 1H, H(6″)], 7.71 [s, 2H, H(2′), H(6′)], 9.7 [s, 1H, CH=N], 11.1 [s, 1H, NH-N], 11.2 [s, 1H, OH]; ^13^C-NMR (100 MHz, DMSOd_6_): *δ* 170.5, 168.4, 163.3, 159.1, 137.4, 132.06 (3C), 132.0, 131.2 (3C), 132.06, 131.3, 129.3 (2C), 128.3 (4C), 121.3 (2C), 117.0, 105.6 (2C), 59.3, 43.6, 39.4, 26.3, 14.9 and 11.7; LC-MS (ESI): [M+H]^+^ 513.40; Anal. Calc. for C_25_H_26_BrN_3_O_4_: C, 58.37; H, 5.11, Br, 15.59, N, 8.20; O, 12.49. Found: C, 58.95; H, 5.09, Br, 15.79, N, 8.25.

(E)-Ethyl 5-(4-bromophenyl)-1-(4-(2-(2-fluorobenzylidene)hydrazinyl)-4-oxobutyl)-2-methyl-1H-pyrrole-3-carboxylate (**15c**)

Yield: 89%; m.p. 141.9–143.2; IR (cm^−1^): 3201 (NH), 2977 (CH_3_ and CH_2_), 1697 (COOC_2_H_5_), 1668 (Amide I), 1566 (Amide II), 1224 (C-O), 814 (p-substituted C_6_H_4_), 519 (C-Br); ^1^H-NMR (*δ*H, 400 MHz, DMSOd_6_): 1.25 [s, 3H, CH_2_CH_3_], 2.07 [t, 2H, CH_2_CH_2_CH_2_], 2.32 [s, 2H, CH_2_CH_2_CH_2_], 2.51 [s, 3H, CH_3_(2)], 4.18 [s, 2H, N(CH_2_)], 4.25 [s, 2H, CH_2_CH_3_], 6.4 [s, 1H, H(4)], 7.28 [s, 2H, H(3″)], 7.36 [s, 2H, H(5″)], 7.52 [s, 1H, H(4″)), 7.69 [s, 2H, H(3′), H(5′)], 7.76 [s, 2H, H(2′), H(6′)], 7.86 [s, 1H, H(6″)], 8.54 [s, 1H, CH=N], 11.4 [s, 1H, NH-N]; ^13^C-NMR (100 MHz, DMSOd_6_): *δ* 170.4, 168.5, 163.2, 159.6, 143.3, 132.06 (2C), 132.6, 132.0, 131.2 (2C), 130.8, 124.4, 115.6, 118.2, 105.6, 104.9, 59.3, 43.6, 39.4, 26.3, 14.9 and 11.4; LC-MS (ESI): [M+H]^+^ 515.40.; Anal. Calc. for C_25_H_25_BrN_3_O_3_F: C, 58.37; H, 4.90, Br, 15.53, N, 8.17; O, 9.33; F, 3.69. Found: C, 58.95; H, 5.09, Br, 15.79, N, 8.25; F, 3.69.

(E)-Ethyl 5-(4-bromophenyl)-1-(4-(2-(3-fluorobenzylidene)hydrazinyl)-4-oxobutyl)-2-methyl-1H-pyrrole-3-carboxylate (**15d**).

Yield: 90%; m.p. 134.1–135.9; IR (cm^−1^): 3208 (NH), 2979 (CH_3_ and CH_2_), 1669 (COOC_2_H_5_), 1625 (Amide I), 1582 (Amide II), 1247 (C-O), 827 (p-substituted C_6_H_4_), 516 (C-Br); ^1^H-NMR (*δ*H, 400 MHz, DMSOd_6_): 1.26 [s, 3H, CH_2_CH_3_], 2.06–2.08 [m 2H, CH_2_CH_2_CH_2_], 2.20 [s, 2H, CH_2_CH_2_CH_2_], 2.49–2.54 [m, 3H, CH_3_(2)], 3.95 [s, 2H, N(CH_2_)], 4.20 [s, 2H, CH_2_CH_3_], 6.44 [s, 1H, H(4)], 7.36 [s, 2H, H(4″)], 7.60 [s, 1H, H(6″)], 7.63 [s, 2H, H(5″)], 7.68–7.69 [m, 2H, H(3′), 7.75 [s, 2H, H(2′), H(6′)], 7.80 [s, 1H, H(2″)), H(5′)], 8.7 [s, 1H, CH=N], 11.4 [s, 1H, NH-N]; ^13^C-NMR (100 MHz, DMSOd_6_): *δ* 173.4, 164.0, 161.1, 161.6, 136.7, 136.5, 132.04 (2C), 132.3, 131.2, 125.2, 125.3 (2C), 118.6, 114.7 (2C), 51.5, 40.6, 39.4, 25.7, 20.8 and 19.7; LC-MS (ESI): [M+H]^+^ 515.40; Anal. Calc. for C_25_H_25_BrN_3_O_3_F: C, 58.37; H, 4.90, Br, 15.53, N, 8.17; O, 9.33; F, 3.69. Found: C, 58.45; H, 4.95, Br, 15.49, N, 8.17; F, 3.71.

(E)-Ethyl 5-(4-bromophenyl)-1-(4-(2-(4-methoxybenzylidene)hydrazinyl)-4-oxobutyl)-2-methyl-1H-pyrrole-3-carboxylate (**15e**).

Yield: 73%; m.p. 133.0–134.4; IR (cm^−1^): 3234 (NH), 2956 (CH_3_ and CH_2_), 1689 (COOC_2_H_5_), 1669 (Amide I), 1567 (Amide II), 1245 (C-O), 832 (p-substituted C_6_H_4_), 546 (C-Br); ^1^H-NMR (*δ*H, 400 MHz, DMSOd_6_): 1.25–1.27 [m, 3H, CH_2_CH_3_], 2.03–2.06 [m, 2H, CH_2_CH_2_CH_2_], 2.49–2.50 [m, 3H, CH_3_(2)], 2.51–2.54 [m, 2H, CH_2_CH_2_CH_2_], 3.94–4.04 [m, 2H, N(CH_2_)], 4.15–4.20 [s, 2H, CH_2_CH_3_], 3.8 [s, 3H, OCH_3_], 6.4 [s, 1H, H(4)], 7.04–7.07 [m, 2H, H(2″), H(6″)], 7.34–7.38 [m, 2H, H(3′), H(5′)], 7.61–7.63 [s, 2H, H(2′), H(6′)], 7.83–7.84 [m, 2H, H(2″), H(6″)], 8.6 [s, 1H, NH-N], 9.7 [s, 1H, CH=N]; ^13^C-NMR (100 MHz, DMSOd_6_): *δ* 170.5, 168.4, 163.3, 159.1, 137.4, 132.06 (2C), 132.0, 131.2 (2C), 132.06, 131.3, 129.3 (2C), 128.3 (2C), 121.3 (2C), 117.0, 105.6 (2C), 59.3, 43.6, 39.4, 26.3, 14.9 and 11.7; LC-MS (ESI): [M+H]^+^ 527.43; Anal. Calc. for C_26_H_28_BrN_3_O_4_: C, 59.32; H, 5.36, Br, 15.18, N, 7.98; O, 12.16. Found: C, 59.95; H, 5.09, Br, 15.18, N, 7.95.

(E)-Ethyl 5-(4-bromophenyl)-1-(4-(2-(4-(dimethylamio)benzylidene)hydrazinyl)-4-oxobutyl)-2-methyl-1H-pyrrole-3-carboxylate (**15f**).

Yield: 69%; m.p. 131.7–132.9; IR (cm^−1^): 3266 (NH), 2979 (CH_3_ and CH_2_), 1682 (COOC_2_H_5_), 1602 (Amide I), 1521 (Amide II), 1242 (C-O), 809 (p-substituted C_6_H_4_), 544 (C-Br); ^1^H-NMR (*δ*H, 400 MHz, DMSOd_6_): 1.26 [s, 3H, CH_2_CH_3_], 2.2 [s, 2H, CH_2_CH_2_CH_2_], 2.50 [m, 3H, CH_3_(2)], 2.57 [s, 2H, CH_2_CH_2_CH_2_], 3.6 [s, 6H, N(CH_3_)2], 4.04 [m, 2H, N(CH_2_)], 4.19 [s, 2H, CH_2_CH_3_], 6.4 [s, 1H, H(4)], 6.81 [s, 2H, H(2″), H(6″)], 7.50 [s, 2H, H(2″), H(6″)], 7.62–7.67 [m, 2H, H(3′), H(5′)], 7.71–7.73 [s, 2H, H(2′), H(6′)], 8.54 [s, 1H, CH=N], 8.0 [s, 1H, NH-N]; ^13^C-NMR (100 MHz, DMSOd_6_): *δ* 191.0, 170.5, 168.4, 163.3, 146.8, 137.0, 135.4, 134.2, 132.0, 131.2 (2C), 129.3, 129.1 (2C), 128.3 (2C), 121.3 (2C), 117.0, 105.6 (2C), 59.3, 43.6, 39.4, 26.3, 14.9 and 11.7; LC-MS (ESI): [M+H]^+^ 532.36.; Anal. Calc. for C_23_H_23_BrN_4_O_6_: C, 51.99; H, 4.36, Br, 15.04, N, 10.54; O, 18.07. Found: C, 51.95; H, 4.42, Br, 15.18, N, 10.59.

(E)-Ethyl 5-(4-bromophenyl)-1-(4-(2-(3-formylbenzylidene)hydrazinyl)-4-oxobutyl)-2-methyl-1H-pyrrole-3-carboxylate (**15g**).

Yield: 55%; m.p. 136.6–139.4; IR (cm^−1^): 3198 (NH), 2931 (CH_3_ and CH_2_), 1695 (COOC_2_H_5_), 1601 (Amide I), 1523 (Amide II), 1247 (C-O), 813 (p-substituted C_6_H_4_), 532 (C-Br); ^1^H-NMR (*δ*H, 400 MHz, DMSOd_6_): 1.25 [s, 3H, CH_2_CH_3_], 2.04–2.07 [m 2H, CH_2_CH_2_CH_2_], 2.23 [s, 2H, CH_2_CH_2_CH_2_], 2.37–2.42 [m, 3H, CH_3_(2)], 4.06 [s, 2H, N(CH_2_)], 4.20 [s, 2H, CH_2_CH_3_], 6.4 [s, 1H, H(4)], 7.36 [s, 2H, H(4″)], 7.60 [s, 1H, H(6″)], 7.63 [s, 2H, H(5″)], 7.68–7.69 [m, 2H, H(3′), 7.75 [s, 2H, H(2′), H(6′)], 7.80 [s, 1H, H(2″)), H(5′)], 9.88 [s, 1H, CHO], 8.0 [s, 1H, CH=N], 11.1 [s, 1H, NH-N]; ^13^C-NMR (100 MHz, DMSOd_6_): *δ* 191, 173.4, 164.0, 161.1, 146.8, 137.7, 135.0, 132.04 (2C), 132.3, 129.3, 129.1 (2C), 125.2, 118.6, 114.7 (2C), 51.5, 40.6, 39.4, 25.7, 20.8 and 19.7; LC-MS (ESI): [M+H]^+^ 540.47.; Anal. Calc. for C_27_H_31_BrN_4_O_3_: C, 60.11; H, 5.79, Br, 14.81, N, 10.39; O, 8.90. Found: C, 59.95; H, 5.69, Br, 14.18, N, 10.45.

### 4.2. Molecular Docking Simulations

Molecular docking was performed using Glide (Schrödinger) by applying the most precise docking mode, XP docking. The crystal structure of MAO-B was retrieved from the Protein Data Bank (PDB) with a co-crystallized ligand (PDB:2V5Z). The protein was refined using the Protein Preparation module of Maestro. The corresponding hydrogen bonds were added, and the defined het states were generated. The processing continued with the minimization of the X-ray structure using the OPLS4 force field. The grid box was set around the co-crystallized ligand, safinamide. The chemical structures of **11**, **12**, and **15** were drawn using the 2D sketcher module in Maestro and converted to the corresponding three-dimensional (3D) structures using the Ligprep module (Schrödinger Release 2024-1: LigPrep, Schrödinger, LLC, New York, NY, USA, 2024). Hydrogen bonds were generated, and energy minimization was performed by applying the OPLS4 force field. The docking protocol was previously validated using redocking procedures [47].

### 4.3. Animals

Wistar rats (20 per group) were obtained from the National Breeding Center of the Bulgarian Academy of Sciences (Sofia, Bulgaria). They were kept under standard conditions in plexiglass cages with free access to water and food and a 12/12 h light/dark regime at 20–25 °C. Food was withdrawn 12 h before each study. The experiments were conducted in accordance with Ordinance No. 15 on Minimum Requirements for the Protection and Welfare of Experimental Animals (SG No. 17, 2006), the European Regulation for the Handling of Experimental Animals, and Permission No. 304, valid until 28 June, 2026, from the Bulgarian Food Safety Agency.

### 4.4. Preparation of Rat Brain Synaptosomes and Mitochondria

Synaptosomes and mitochondria were obtained by multiple subcellular fractionation using a Percoll gradient [48].

For the planned in vitro evaluations, the isolated fractions were incubated with the test substances at a concentration of 50 µM for 1 h.

#### 4.4.1. Establishing a Dopamine Model of Neurotoxicity

This in vitro model resembles the neurodegenerative processes that primarily occur in PD. The metabolism of 6-OHDA leads to the production of reactive quinones (*p*-quinone), which in turn leads to the formation of ROS. Reactive metabolites and ROS damage the pre- and post-synaptic membrane, leading to neuronal cell damage [49]. Synaptosomes were incubated with 6-OHDA (150 μM) for 1 h.

#### 4.4.2. MTT Assay to Assess Synaptosomal Viability

After 1 h of incubation with 50 µM of the tested substances and 150 μM of the toxic agent, synaptosomes were centrifuged and washed twice with the buffer to remove 6-hydroxydopamine, which can interact with MTT. For the ‘washed’ synaptosomes, 60 µL of MTT solution was added. The plates were incubated with MTT solution at 37 °C for 10 min. After incubation, the samples were centrifuged at 15,000× *g* for 2 min. The excess liquid was removed, and a DMSO solution was used to dissolve the formed formazan crystals. After dissolution, the amount of formazan was measured spectrophotometrically at λ = 580 nm [50].

#### 4.4.3. Determination of Reduced Glutathione (GSH) in Isolated Brain Synaptosomes by the Method of Robyt [51]

After incubation, the synaptosomes were centrifuged at 4000× *g* for 3 min. The supernatant was removed, and the pellet was used for GSH determination. It was treated with 5% trichloroacetic acid, left for 10 min on ice, and centrifuged at 8000× *g* for 10 min (2 °C). The supernatant was collected for GSH determination and frozen at −20 °C. Immediately before measurement, the samples were neutralized with 5N NaOH [51].

### 4.5. Tert-Butyl Hydroperoxide-Induced Oxidative Stress

Isolated rat brain mitochondria were incubated with 75 µM tert-butyl hydroperoxide (*t*-BuOOH) [52].

#### 4.5.1. Determination of Malondialdehyde (MDA) Production in Brain Mitochondria [53]

To the mitochondria, 0.3 mL of 0.2% thiobarbituric acid and 0.25 mL of sulfuric acid (0.05 M) were added, and the mixture was boiled for 30 min. After boiling, the tubes were placed on ice, and 0.4 mL of *n*-butanol was added to each tube, which was then centrifuged at 3500× *g* for 10 min. The amount of MDA was determined spectrophotometrically at 532 nm.

#### 4.5.2. Determination of GSH Level in Brain Mitochondria [53]

After incubating the mitochondria with the substances and tert-butyl hydroperoxide, the reaction was stopped with 5% trichloroacetic acid, and each sample was homogenized with the acid and left on ice. After centrifugation of the homogenate at 6000× *g*, a 0.04% solution of DTNB was added to the supernatant to give a yellow color, and the absorbance was measured at 412 nm.

### 4.6. Isolation of Brain Microsomes [54]

The brain was homogenized in nine volumes of 0.1 M Tris buffer containing 0.1 mM dithiothreitol, 0.1 mM phenylmethylsulfonyl fluoride, 0.2 mM EDTA, 1.15% KCl, and 20% (*v*/*v*) glycerol (pH 7.4). The resulting homogenate was centrifuged twice at 17,000× *g* for 30 min. The supernatants from the two centrifugations were pooled and centrifuged twice at 100,000× *g* for 1 h. The pellet was then frozen in 0.1 M Tris buffer.

### 4.7. Iron/Ascorbate-Induced Lipid Peroxidation (LPO)

Non-enzyme-induced lipid peroxidation was induced using 20 μM ferrous sulfate solution and 0.5 mM ascorbic acid solution [55].

#### Determination of MDA in Brain Microsomes [55]

After incubation of the microsomes with the substances and toxic agents, the reaction was stopped by the addition of 0.5 mL of 20% trichloroacetic acid, followed by 0.5 mL of 0.67% thiobarbituric acid. The ongoing reactions are associated with the formation of a colored complex between the formed malondialdehyde and the added thiobarbituric acid. MDA was determined spectrophotometrically at 535 nm. A molar extinction coefficient of 1.56 × 105 M-1 cm^−1^ was used for the calculation.

### 4.8. Determination of Human Recombinant MAOA/B Enzyme Activity

Working solutions of the test substances, reagents, and human recombinant MAOA/B enzyme (*h*MAOA/B) were prepared in a reaction buffer according to the manufacturer’s instructions. The substances were applied at a final concentration of 1 µM. The substances, together with *h*MAOA/B, were placed in a 96-well plate (eight samples for each substance), and the plate was placed in an incubator for 30 min (in the dark, at 37 °C). The activity of recombinant human MAOA/B was determined fluorimetrically at two wavelengths (570 nm and 690 nm) with fluorimetric readings performed in a Synergy 2 Microplate Reader. Tyramine hydrochloride was used as the substrate. The activity was determined by detecting H_2_O_2_ production. This production is reported by binding to horseradish peroxidase using *N*-acetyl-3,7-dihydroxyphenoxazine (AmplexRed, Thermo Fisher Scientific, Waltham, MA, USA) [56].

### 4.9. Histopathological Analysis

The samples from mouse brains were fixed in 10% buffered neutral formalin solution. The fixed materials were dehydrated in ascending grades of alcohol: 50°, 60°, 70°, 80°, 90°, 96°, and absolute alcohol. After dehydration, the samples were cleared with xylene and embedded in paraffin blocks. Sections with a thickness of 5 μm were prepared using a rotary microtome. The sections were attached to slides using histological glue, processed with xylene, rehydrated using a descending alcohol row–absolute alcohol, 96°, 90°, 80°, 70°, 60°, and 50°, and finally stained with Hematoxylin-Eosin [57]. Microscopic examination and photography were performed using a Levenhuk D740T light microscope with an integrated camera.

### 4.10. Statistical Methods

The results of the in vivo evaluations and the experiments performed on isolated rat brain synaptosomes, mitochondria, and microsomes were statistically processed using the ‘MEDCALC’ version 23.3.2 program using the non-parametric Mann-Whitney method at significance levels *p* < 0.05; *p* < 0.01, and *p* < 0.001.

The results obtained from *h*MAOA/B activity were statistically processed using GraphPad Prism software (version 5.0).

## 5. Conclusions

A clearer understanding of MAOB’s role in Parkinson’s may help optimize the use of MAOB inhibitors and support the development of new therapies that more precisely target its activity or expression. Thus, a group of *N*-pyrrolylcarbohydrazide hydrazone derivatives were synthesized using classical Paal-Knorr cyclocondensation. The compounds were obtained in good yields (55–90 %). Their structures were elucidated based on the corresponding spectral characteristics, and their purity was proven through TLC, melting points, elemental analysis, and MS data. The binding capacity of the initial hydrazide was determined using molecular docking. Overall, the virtual evaluation showed that the prolongation of the methylene linker could be considered a reason for the deterioration of the degree of binding to the active site of the enzyme, leading to reduced inhibitory effects against MAOB. In contrast, extended pharmacological and toxicological studies on subcellular fractions (isolated rat brain synaptosomes, mitochondria, and microsomes) of the evaluated target hydrazones showed that when applied alone, all substances showed weak neurotoxic effects relative to the control (untreated subcellular fractions), with **15a**, **15d**, and **15f** being the least toxic representatives. In addition, the corresponding neuroprotective effects of the aforementioned least toxic molecules were determined using three in vitro models of oxidative damage. The results indicated that **15d** was the best protector. All newly synthesized molecules were tested for possible MAOA and/or MAOB inhibitory effects. The results indicated that, except for **15f**, all other tested substances at a concentration of 1 µM did not exhibit inhibitory activity on MAOA but exhibited good inhibitory effects on MAOB, with **15d** presenting the best *h*MAOB selectivity index (>471); thus, it was considered a potential selective MAOB inhibitor. The latter was subjected to an in vivo Rotenone-induced model of Parkinsonism in mice for possible neuroprotection. The results indicated good protective effects of **15d**, making it the most promising derivative for further evaluation.

## Figures and Tables

**Table 1:** IDs, melting points (°C), TLC characteristics (R_f_), MS data, and yields for the new hydrazones.

IDs	m.p.	R_f_	MS Data [M + H]^+^ (*m*/*z*)	Yields%
13	93.4–94.8 °C	0.83	395.26	88
15	99.8–101.5 °C	0.56	409.30	81
15a	110.0–110.9 °C	0.50	497.40	83
15b	119.5–120.3 °C	0.53	513.40	87
15c	141.9–143.2 °C	0.60	515.40	89
15d	134.1–135.9 °C	0.60	515.40	90
15e	133.0–134.4 °C	0.56	527.43	73
15f	131.7–132.9 °C	0.58	532.36	69
15g	136.6–137.4 °C	0.60	540.47	55

**Table 2:** IC_50_ (EC_50_) of the newly synthesized hydrazones **15a**, **15d**, **15f**, Selegiline, and Chlorgyline on *h*MAOA/B.

Compounds	IC_50_ (EC_50_), (µM ± SD) *h*MAOA	IC_50_ (EC_50_), (µM ± SD) *h*MAOB	SI
**15a**	>200	0.692	>289
**15d**	>200	0.425	>471
**15f**	0.821	0.710	1.16
Selegiline	-	0.320 ± 0.20	
Chlorgyline	18.74 ± 0.096	-	

## Data Availability

The original contributions presented in this study are included in the article. Further inquiries can be directed to the corresponding author Maya Georgieva.
